# Valorization of *Spirodela polyrrhiza* biomass for the production of biofuels for distributed energy

**DOI:** 10.1038/s41598-023-43576-y

**Published:** 2023-10-02

**Authors:** Z. Romanowska-Duda, K. Piotrowski, S. Szufa, M. Sklodowska, M. Naliwajski, C. Emmanouil, A. Kungolos, A. A. Zorpas

**Affiliations:** 1 Department of Plant Ecophysiology, University of Lodz, Banacha Str. 12/16, 92-237 Lodz, Poland; 2 Faculty of Process and Environmental Engineering, Lodz University of Technology, Wolczanska 213, 90-924 Lodz, Poland; 3 Department of Plant Physiology and Biochemistry, University of Lodz, Banacha Str. 12/16, 92-237 Lodz, Poland; 4 Department of Planning and Regional Development, Aristotle University of Thessaloniki, Thessaloniki, Greece; 5 Civil Engineering Department, Aristotle University of Thessaloniki, Thessaloniki, Greece; 6 Laboratory of Chemical Engineering and Engineering Sustainability, Faculty of Pure and Applied Sciences, Open University of Cyprus, Giannou Kranidioti 89, Latsia, 2231 Nicosia, Cyprus

**Keywords:** Physiology, Plant sciences

## Abstract

Considering the main objectives of a circular economy, *Lemnaceae* plants have great potential for different types of techniques to valorize their biomass for use in biofuel production. For this reason, scientific interest in this group of plants has increased in recent years. The aim of this study was to evaluate the effects of salt stress on the growth and development of *S. polyrrhiza* and the valorization of biomass for biofuel and energy production in a circular economy. Plants were grown in a variety of culture media, including standard 'Z' medium, tap water, 1% digestate from a biogas plant in Piaszczyna (54° 01′ 21″ N, 17° 10′ 19″ E), Poland) and supplemented with different concentrations of NaCl (from 25 to 100 mM). Plants were cultured under phytotron conditions at 24 °C. After 10 days of culture, plant growth, fresh and dry biomass, as well as physio-chemical parameters such as chlorophyll content index, gas exchange parameters (net photosynthesis, transpiration, stomatal conductance and intercellular CO_2_ concentration), chlorophyll fluorescence measurements were analyzed. After 10 days of the experiment, the percentage starch content of *Spirodela* shoot segments was determined. *S. polyrrhiza* was shown to have a high starch storage capacity under certain unfavorable growth conditions, such as salt stress and nutrient deficiency. In the W2 (50 mM NaCl) series, compared to the control (Control2), starch levels were 76% higher in shoots and 30% lower in roots. The analysis of the individual growth and development parameters of *S. polyrrhiza* plants in the experiment carried out indicates new possibilities for the use of this group of plants in biofuel and bioethanol production.

## Introduction

The increasing demand for alternative energy sources has caused a significant development of technologies for obtaining renewable fuels derived from biological raw materials. Reduction of greenhouse gas emissions, biodegradability, diversification in fuel sectors, development of the agricultural products market and sustainable development are the main reasons for increasing the expenditure on obtaining renewable energy from liquid fuels such as bioethanol and biodiesel. On the other hand, bio-waste treatment processes, including wastewater treatment plants, biogas plants and composting, should be adopted to prevent bio-waste from going to landfills, protect the environment and comply with European environmental policy^1,2^, and, if possible, convert it into biofuels and renewable energy to reduce climate change^3^. Using different pre-treatment techniques we can valorize specific biomass for specific purposes–for example the torrefaction process can significantly increase the amount of carbon in solid biofuel^4^.

Starch next to cellulose is one of the most common biopolymers on earth. It is not only one of the main elements of the everyday carbohydrate diet of every person, but above all it is a very important substrate used for food, paper, pharmaceutical, and textiles. In recent years it is used in the energy industry for the production of fuels such as bioethanol^5^. Starch is an insoluble polysaccharide, a polymer of glucose residues synthesized in chloroplasts of higher plants, acting as a storage of energy generated during photosynthesis^6^. This sugar is produced during the day in plant organs characterized by high photosynthesis intensity, and then used at night to support continuous metabolism, therefore starch is considered as one of the basic factors regulating plant growth^7^. In addition, starch plays a key role in plant responses to abiotic stress caused by water scarcity, high environmental salinity or adverse extreme temperatures. Under unfavorable conditions, many biochemical processes are limited, including photosynthesis, which mobilizes starch storage and then its break-down to allow normal plant growth. These sugars also have an initiating function and along the signaling pathways together with abscisic acid (ABA) enabling plant reaction to stress^8^. Starch degradation in response to stress often correlates with tolerance to these conditions^9^. Gonzalez-Cruz and Pastenes (2012)^10^ proved that a drought-resistant variety of bean (*Phaseolus vulgaris*) degraded more starch than a drought-sensitive one^11^. In addition, Cuellar-Ortiz et al.^12^ reported that increased accumulation of carbohydrates occurred only in the drought-resistant soybean (*Glycine max*). It was shown that various factors, including deficiency of macro- and microelements, salt stress, pH, temperature and photoperiod might increase the carbohydrate content in plant cells^13,14^. These factors reduced photosynthesis and increased production of stored compounds that can ensure plant survival due to increased tolerance of extreme environmental conditions^13^.

Research to date has shown that *Lemnaceae* aquatic plants can be an ideal source of starch for the energy industry^15,16^. *Lemnaceae* are common monocotyledonous aquatic plants also called water duckweed, the most popular are: *Spirodela*, *Landoltia*, *Lemna*, *Wolffiella* and *Wolffia*^17,18^. These macrophytes are precursors of new generation crops, their cultivation is relatively easy and cost effective^18,19^. Fast growth and high accumulation of starch are two key features of the duckweed potential as a raw material for biofuel production. Duckweed grows faster than most other plants and under favorable conditions it can double its biomass within 23–24 hours^20^. According to Ma et al. (2018) and Cheng et al. (2017) the starch content in *Lemnaceae* varies depending on the species and growth conditions, from 3 to 60% of dry matter^21,22^. The macrophytes can produce starch in an amount of about 28 t ha^−1^ year^−1^, compared to corn starch production of about 5.0 t ha^−1^ year^−1^^23^. The ethanol yield from 1 ha of duckweed is about 50% higher than in the case of corn-based ethanol^16^. High starch storage by plants (*Lemna minor*) (10–36%, m/m) was caused by lack of nutrients or lack of light. The collected duckweed biomass (from wastewater culture) contained 20.3% (w/w) total glucan, 32.3% (w/w) protein, trace hemicellulose and undetectable amounts of lignin^24,25^.

Sodium chloride (NaCl) is the most common salt on earth, both of natural and anthropogenic origin. Stress caused by salinity of the environment limits the growth and development of terrestrial plants and degrades freshwater flora^26^. In plant cells, osmotic stress induced by NaCl is responsible for the formation of reactive oxygen species (ROS), the resulting oxidative stress leads to degradation of many cellular macromolecules^27,28^. However, sodium chloride is the simplest and cheapest stress factor to be used for the effective production of biomass and biofuels on an industrial scale^29^. Carbohydrates play a key role in the carbon economy of plants, in photosynthesis, homeostasis and lipid metabolism^30^. Reduction of carbohydrate content in plant tissues may be caused by decrease in photosynthesis intensity, inhibition of cell division and osmotic imbalance which occur during severe salt stress^13,14,31^. This stress triggers intensive starch storage in various parts of *Lemnaceae* plants, which makes this plant very good raw material for the production of biofuels^31,32^.

*Spirodela polyrrhiza* is one of the most popular species of duckweed, inhabiting fresh water ecosystems. Great potential for the production and accumulation of starch under various growth conditions is characteristic of this plant^29,33^. The literature data indicate that macrophytes are involved in phytoremediation, as they take up heavy metals from polluted waters and improve the overall quality of water reservoirs^19,34^. Wendeou et al. (2013) and Romanowska-Duda et al. (2019) pointed out that high expansion of *Spirodela sp*. in contaminated water bodies means that macrophytes, due to their phytoremediation properties, can be successfully used in the process of water purification and biomass production on an industrial scale.

Starch is the best raw material for the production of bioethanol due to the relatively simple conversion process^35^. Basar et al. (2020) explored the impacts of varying doses of cellulase and β-glucosidase enzymes on the production of ethanol and methane from switchgrass. Achieved an increase in energy potential up to 90.6%^36^. Currently, corn is the most popular raw material used in this process, but it is the basic source of food in the world and is mainly grown for the production of feed and food. According to Chang et al. (2012), water duckweed may be an alternative solution replacing corn in the bioethanol production process. It is simply and quickly hydrolyzed and fermented to ethanol with a yield much higher than that of corn^37^. After enzymatic hydrolysis, the ethanol yield obtained from duckweed reached 6.42 × 10^3^ L/ha^−1^, about 50% more than in the case of corn-based ethanol production, which makes *Lemnaceae* a competitive source of starch for the production of ethanol fuel^16^. When the macrophytes were subjected to so-called food stress (for 5–10 days) 31.0–45.8% starch content was observed in their dry matter, and up to 94.7% of starch can be converted into ethanol using existing corn starch conversion technologies^32,38^. In order to increase the amount of carbon content, we can use steam for obtaining better quality of bioethanol. Superheated steam production technology with simultaneous pressure and temperature control of SHS, which contributes to the development of a thermochemical conversion of biomass that is cheaper in industrial application. Precise control affects the safety of the process, i.e. limiting potential fire or explosion. The torrefaction methods used so far are not able to recover valuable by-products of the entire process^39^. Therefore, thanks to the use of SHS for torrefaction processes, it is possible to recover by-products and at the same time valuable products, such as, for example, formic acid and acetic acid. This method, thanks to the cessation of the combustion of products and the optimization of obtaining plant, forest and agricultural biomass, leads to a positive impact on the quality of environmental performance^40^. Economic profitability depends on the use of low-temperature waste heat for pre-drying the biomass and the use of steam instead of other gases such as fossil fuels or gases generated during the torrefaction process, the so-called "Torgas"^41–44^.

Reactor design of the torrefaction process: Lack of economically/environmentally efficient technological options for reactor heating and pre-drying of biomass, use of “torgas” in combination with fossil fuels. Current reactor technologies are inefficient, as they require long residence time of biomass in the reactor Innovative Superheated steam (SHS)-powered torrefaction reactor: improving process control of temperature, torgas production and residence time, reducing residence time and emissions, increasing the recovery rate of by-products from condensate, improving thermal efficiency (heat recovery during water re-condensation), and allowing the use of semi-dry biomass residues (moisture content up to 60%)^45–47^.

By-products from the torrefaction process: Limited knowledge of valorization options, in particular for the wastewater resulting from the condensate, which is rich in volatile organic compounds. Valorization of the condensate by-product for biofuel production (biogas, biohydrogen) and for recovery of bioproducts^48,49^.

The purpose of the experiment was to examine the impact of NaCl on the starch storage process in *S. polyrrhiza* and its possible use for the production of bioethanol.

## Materials and methods

Plant collection, culture, exposure conditions and experiment variants. The experiment was conducted for 10 days under laboratory conditions on the model water plant *S. polyrrhiza*, derived from the collection of in vitro cultures of the Department of Plant Ecophysiology of the Faculty of Bioscience of the University of Lodz, Poland. Plant samples for testing were collected and evaluated according to methodologies developed on the basis of national standards and previous research, which are in force at University of Lodz in Poland and have been used in previous studies and international publication^19^. Test were conducted in accordance with OECD 221 Assess the toxicity of substances to freshwater aquatic plants of the genus *Lemna* (duckweed).

The plants were cultured in the presence of different concentrations of NaCl and in control variants. Morphological observation was carried out daily throughout the experiment. There were three control variants (I) standard "Z" liquid medium (pH 6.4), (II) tap water (pH 7.0) and (III) tap water supplemented with 1% post-fermentation effluent from a biogas plant in Piaszczyna, Poland (pH 8.1). Medium “Z” is a standard medium for growing in vitro culture plants containing all necessary micro and macro elements. Plants were cultivated for 10 days in a phytotron room at 24 °C, under constant lighting with PHILIPS MASTER TL-D lamps with a power of 2 × 18W/840. The pH of the liquid medium "Z" was determined using a Seven Compact™ S210 pH meter. *Spirodela sp*. were grown in 250 mL Erlenmeyer flasks with 100 mL liquid medium.

### Treatments and experimental design

The experimental medium was prepared on the basis of previous results from a macrophyte culture obtained at the Department of Plant Ecophysiology at the University of Lodz. The experiment was carried out according to the following experimental variants:

Control series:

- Control 1–100 mL standard "Z" medium
- Control 2–100 mL of tap water
- Control 3–99 mL of tap water + 1 mL of fermentation effluent from a biogas plant (1% leachate—the optimal concentration obtained on the basis of previous experiments. This leachate concentration does not require preliminarny plant adaptation )

Different variants of medium supplemented with NaCl:

Figures 1, 2, 3.

**Figure 1:**
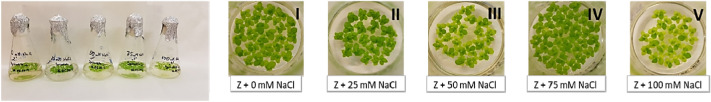
Growth kinetics of *Spirodela polyrrhiza* plants (after 10 days) grown on different medium variants: Control 1–100 mL of standard 'Z' medium (**I**); "Z" medium supplemented with 25 mM NaCl (**II**), "Z" medium supplemented with 50 mM NaCl (**III**), "Z" medium supplemented with 75 mM NaCl (**IV**), "Z" medium supplemented with 100 mM NaCl (**V**).

**Figure 2:**
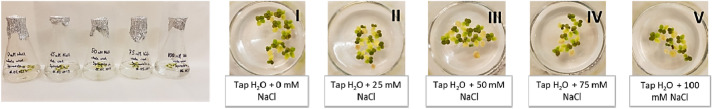
Growth kinetics of *Spirodela polyrrhiza* plants (after 10 days) grown on different nutrient variants: Control with 2–100 mL of tap water (**I**); tap water supplemented with 25 mM NaCl (**II**), tap water supplemented with 50 mM NaCl (**III**), tap water supplemented with 75 mM NaCl (**IV**), tap water supplemented with 100 mM NaCl (**V**).

**Figure 3:**
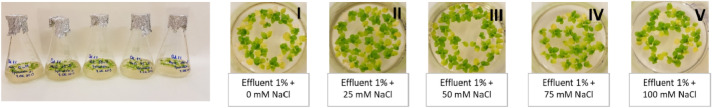
Growth kinetics of *Spirodela polyrrhiza* plants (after 10 days) grown on different nutrient variants: Control 3–99 mL tap water + 1 mL fermentation effluent (**I**); 1% leachate supplemented with 25 mM NaCl (**II**), 1% leachate supplemented with 50 mM NaCl (**III**), 1% leachate supplemented with 75 mM NaCl (**IV**), 1% leachate supplemented with 100 mM NaCl (**V**).

### Assessments of plant physiological activity, growth and chemical properties

On the last day of the experiment, a number of physicochemical analyses were carried out: the index of chlorophyll content in plant leaves was measured using the Minolta SPAD-502 apparatus, Japan; gas exchange parameters i.e. net photosynthesis (mmolH_2_O/m^−2^ s^−1^), transpiration (mmolH_2_O/m^−2^ s^−1^), stomatal conductivity (mmolH_2_O/m^−2^ s^−1^) and intercellular CO_2_ concentration (µmolH_2_O/m^−2^ s^−1^) were marked using a TPS-2 camera (PP Systems, USA). Chlorophyll fluorescence was determined using a specialized Handy PEA fluorimeter from Hansatech Instruments Ltd.

The starch contents in shoot and root tissues (in fresh biomass) collected from the research variants containing increasing NaCl concentrations in the range of 25-100 mM were determined using the specialized Starch Assay Kit from Sigma-Aldrich, product Code STA20. The calculation of the percentage of starch in the samples was made in accordance with the equation indicated by the manufacturer:

1 $$\begin{gathered} \left( {{\text{A blank }} - {\text{ A}}} \right) \times {9}00/\left( {{\text{A standard}} - {\text{A blank}}} \right) \times {\text{tissue weight in mg}} \hfill \\ {\text{A}} - {\text{absorbance at 54}}0{\text{ nm}} \hfill \\ \end{gathered}$$

Equation (1) Formula for calculating the percentage of starch in samples according to the method of the manufacturer Starch Assay Kit from Sigma-Aldrich, product Code STA20.

### Statistical analysis

Statistical analyses were performed with the Statistica 12 program. The data are expressed as mean ± standard deviation (SD) of 10 independent experiments. The normality of the data distribution was assessed by Kołmogorov-Smirnov test. The homogenicity of variances was determined using Levene’a test. Differences between samples were analyzed using one way parametric analysis of variance (ANOVA) with the post-hoc Dunnett’s test *p* values ≤ 0.05 were considered significant.

## Results

### Relative growth rate

Daily observation of morphological features, counting of new shoot segments, physical and chemical analyses performed on the last day indicated the varied sensitivity of *S. polyrrhiza* plants grown on the medium supplemented with various concentrations of NaCl and leachate after methane fermentation. RGR (Relative Growth Rate) is shown in Fig. 5I. When analyzing the kinetics of plant growth, all fronds that were visible, regardless of their size, were taken into account. Based on the number of fronds on the "zero" day (start day of the experiment) and the 10th last day, relative growth rate (RGR) was calculated according to the formula:

2 $$\left( {{\text{ln x2 }} - {\text{ ln x1}}} \right)/{\text{t}}$$

x1–is the number of fronds on the zero day of the test, x2–is the number of fronds on the tenth day of the test, t–specifies the number of days of breeding.

Equation 2 Formula for calculating Relative Growth Rate (RGR).

The series run on "Z" medium supplemented with 75 mM NaCl (Z3), with plant growth 7.5% higher than the control (Control1) was the most effective. In the variant with 1% post-fermentation effluent, the number of macrophyte fronds was higher by 15% (O1–25 mM NaCl) and 7% (O2–50 mM NaCl) compared to the control series (Control2). The beneficial effect of NaCl was also observed in the series with tap water alone, where the plants grown on the medium supplemented with 25 mM NaCl (W1) and 50 mM (W2) showed 3–5% better growth compared to Control3.

### Index of chlorophyll content

**Figure 4:**
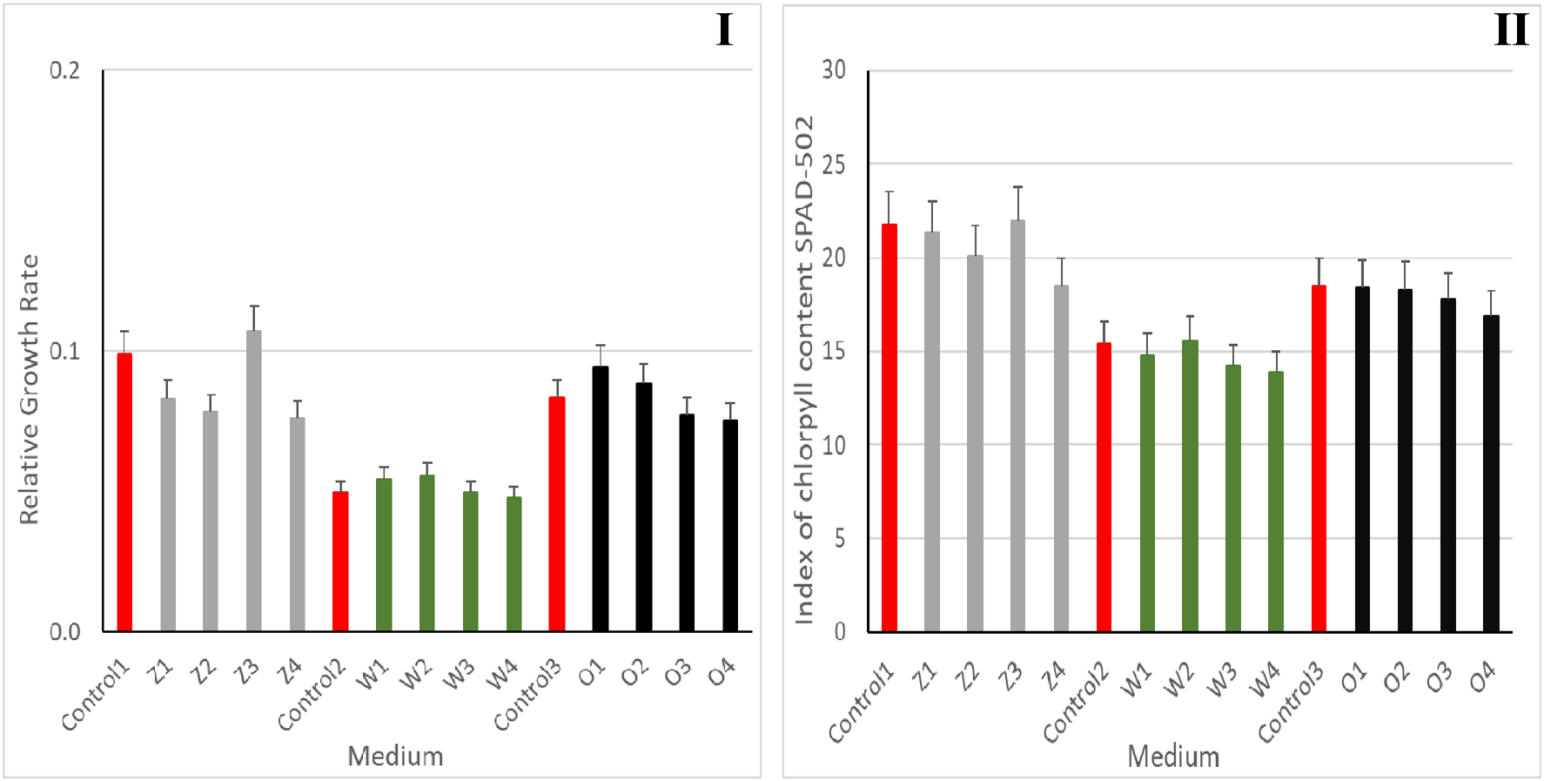
Relative growth rate (RGR) (**I**) and index of chlorophyll content (**II**) in *Spirodela polyrrhiza* plant leaves grown on different variants of NaCl supplemented medium. Control series: Control 1–100 ml of standard "Z" medium; Control 2–100 ml of tap water; Control 3–99 ml tap water + 1 ml post-fermentation effluent; Experimental variants: "Z" medium supplemented with 25 mM NaCl (Z1), "Z" medium supplemented with 50 mM NaCl (Z2), "Z" medium supplemented with 75 mM NaCl (Z3), "Z" medium supplemented with 100 mM NaCl (Z4); tap water supplemented with 25 mM NaCl (W1), tap water supplemented with 50 mM NaCl (W2), tap water supplemented with 75 mM NaCl (W3), tap water supplemented with 100 mM NaCl (W4); 1% leachate supplemented with 25 mM NaCl (O1), water 1% leachate supplemented with 50 mM NaCl (O2), 1% leachate supplemented with 75 mM NaCl (O3), 1% leachate supplemented with 100 mM NaCl (O4). Data are expressed as mean ± SD of 10 experiments; Statistical analyses were performed using one-way parametric Anova test with the post-hoc Dunnett’s test. *p < 0.05% versus control.

Similar results were obtained when analyzing the chlorophyll content index of Fig. 4I,II. The highest concentration of chlorophyll in *Lemnaceae* shoots (fronds) was obtained in the variant with "Z" standard medium supplemented with 75 mM NaCl (Z3). In the other variants the values were similar to the control samples.

### Gas exchange parameters

**Figure 5:**
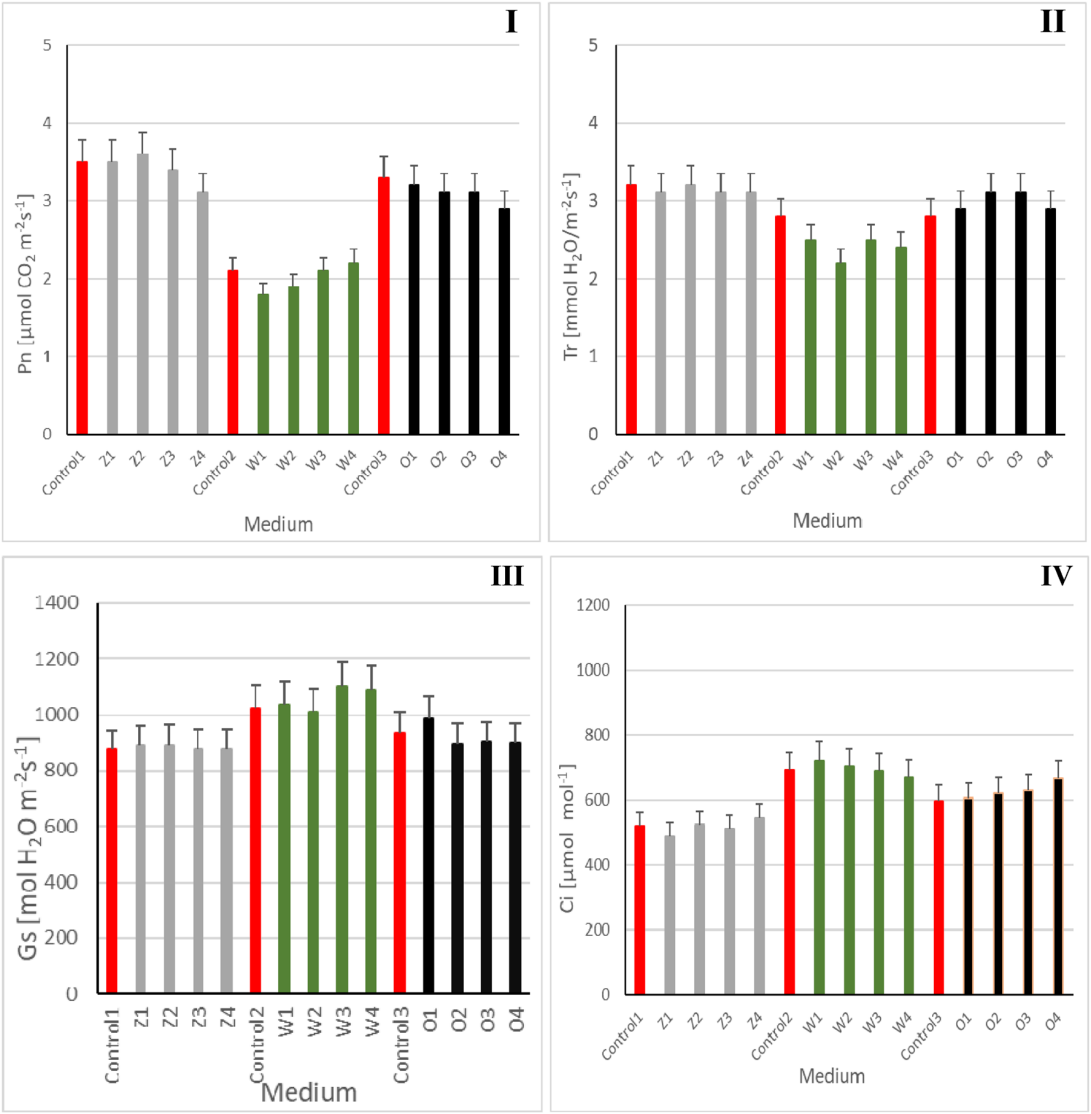
Net photosynthesis (**I**), transpiration (**II**), stomatal conductance (**III**) and intercellular concentration of CO_2_ (**IV**) in leaves of *Spirodela polyrrhiza* plants grown on different variants of NaCl supplemented medium. Control series: Control 1–100 mL of standard "Z" medium; Control of 2–100 mL of tap water; Control 3–99 ml of tap water + 1 mL of digestate; Experimental variants: "Z" medium supplemented with 25 mM NaCl (Z1), "Z" medium supplemented with 50 mM NaCl (Z2), "Z" medium supplemented with 75 mM NaCl (Z3), "Z" medium supplemented with 100 mM NaCl (Z4); tap water supplemented with 25 mM NaCl (W1), tap water supplemented with 50 mM NaCl (W2), tap water supplemented with 75 mM NaCl (W3), tap water supplemented with 100 mM NaCl (W4); 1% leachate supplemented with 25 mM NaCl (O1), water 1% leachate supplemented with 50 mM NaCl (O2), 1% leachate supplemented with 75 mM NaCl (O3), 1% leachate supplemented with 100 mM NaCl (O4). Data are expressed as mean ± SD of 10 experiments; Statistical analyses were performed using one-way parametric Anova test with the post-hoc Dunnett’s test. *p < 0.05% versus control.

On the tenth day of the experiment, several biochemical analyses were carried out, gas exchange parameters were determined, including the intensity of photosynthesis in the fronds of *S. polyrrhiza* plants. In the series of macrophytes grown on "Z" standard medium, the most favorable effect and value of net photosynthesis (Fig. 5I) compared to the control (Control1) was observed in the 50 mM NaCl (Z2) and the 25 mM NaCl (Z1) variants and slightly weaker (by 3%) in the variant with 75 mM NaCl (Z3), and the least favorable (weaker by 13%) with 100 mM NaCl. The value of net photosynthesis in the plants grown on medium with the addition of 1% post-fermentation effluent was characterized by a decrease that was inversely proportional to the concentration used, and so the intensity of the net photosynthesis process compared to the control (Control3) was successively lower in the O1 variant 25 mM NaCl by 3%, in O2 and O3 (50 mM and 75 mM) by 6.5%, and in the O4 series (100 mM NaCl) lower by 13%.

The intensity of the transpiration process observed in all variants grown on “Z” medium (Fig. 5II) remained at a level similar to control (Control1) and ranged from 3 to 3.2 mmolH_2_O/m^2^s^1^. A different tendency was demonstrated by the series grown on the medium with 1% post-fermentation effluent with tap water. Transpiration was characterized by a greater intensity compared to the control (Control2): O1 and O4 variants (25 mM and 100 mM NaCl) higher by 3.5% and O2 and O3 variants (50 and 75 mM NaCl) higher by 10%.

The stomatal conductivity (Fig. 5III) in the variants with the "Z" standard medium was similar and ranged from 876 to 902 molH_2_O/m^2^s^1^. This parameter was different in the series carried out on tap water with the addition of NaCl, variant W1 and W2 (25 and 50 mM NaCl) remained at a similar level as control (Control2) while W3 and W4 (75 and 100 mM NaCl ) were higher than the controls by 7 and 6.5% respectively. In the series with 1% leachate, the values of stomatal conductivity were 5.5% higher O1 (25 mM) and 4.5% lower O2, O3 and O4 (50, 75, 100 mM NaCl compared to the control (Control3).

In contrast, the highest content of intercellular CO_2_ was determined in the series with tap water supplemented with: 25 mM NaCl (W1), 50 mM NaCl (W2), 75 mM NaCl (W3). The values obtained were quite similar and ranged from: 671–721 compared to the control (691), with variant W1 having a higher value than control 2. It should also be noted that all experimental variants containing 'Z' medium had the lowest CO_2_ content values compared to the series containing tap water and 1% leachate, indicating a more efficient photosynthetic process (Fig. 5lV).

### Chlorophyll fluorescence and fresh biomass

**Figure 6:**
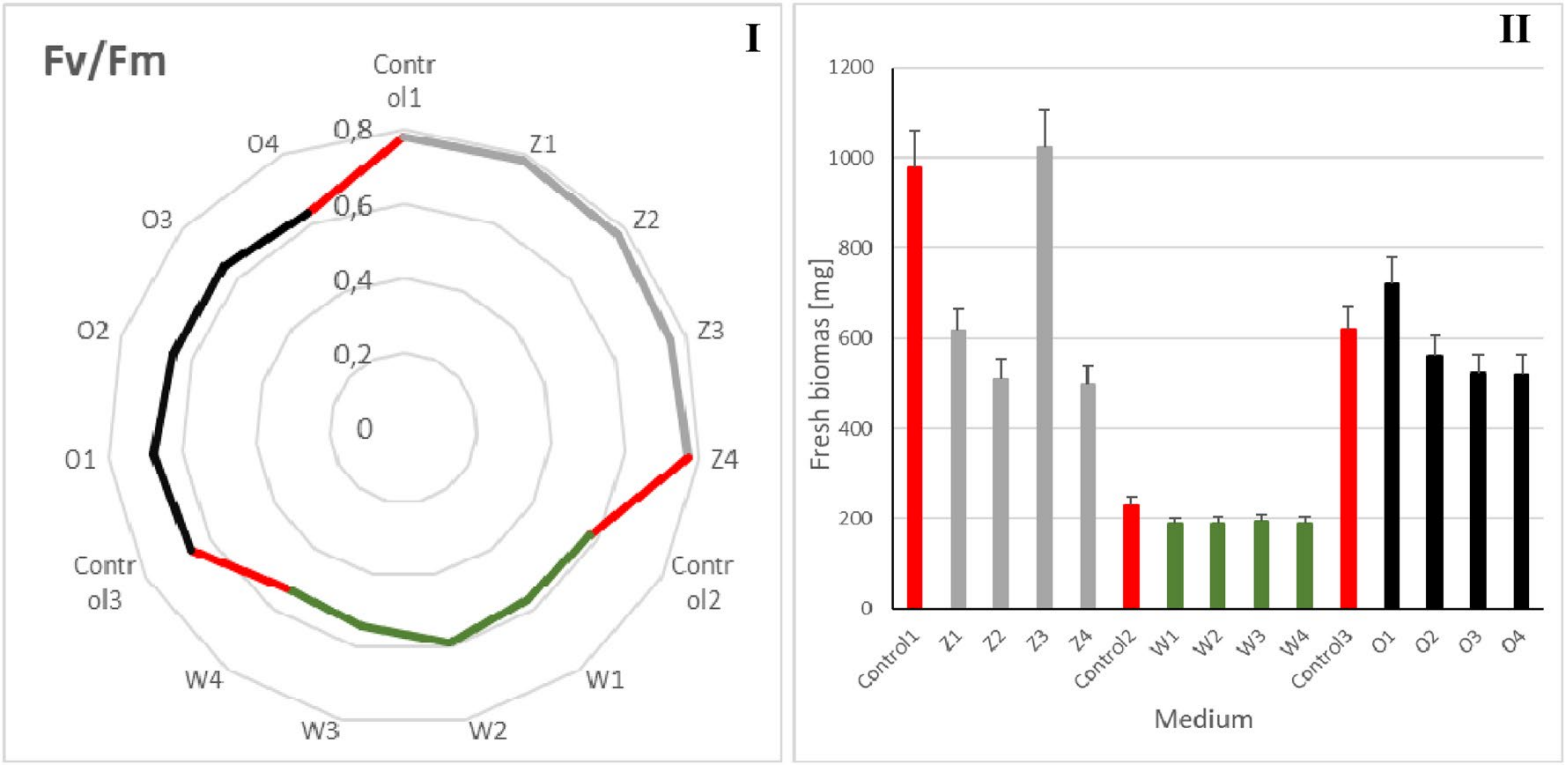
Chlorophyll fluorescence (**I**) and fresh biomass (**II**) of *Spirodela polyrrhiza* plants grown on different variants of NaCl supplemented medium. Control series: Control 1–100 mL of "Z" medium; Control 2–100 mL of tap water; Control 3–99 ml tap water + 1 mL post-fermentation effluent; Experimental variants: "Z" medium supplemented with 25 mM NaCl (Z1), "Z" medium supplemented with 50 mM NaCl (Z2), "Z" medium supplemented with 75 mM NaCl (Z3), "Z" medium supplemented with 100 mM NaCl (Z4); tap water supplemented with 25 mM NaCl (W1), tap water supplemented with 50 mM NaCl (W2), tap water supplemented with 75 mM NaCl (W3), tap water supplemented with 100 mM NaCl (W4); 1% leachate supplemented with 25 mM NaCl (O1), water 1% leachate supplemented with 50 mM NaCl (O2), 1% leachate supplemented with 75 mM NaCl (O3), 1% leachate supplemented with 100 mM NaCl (O4). Data are expressed as mean ± SD of 10 experiments; Statistical analyses were performed using one-way parametric Anova test with the post-hoc Dunnett’s test. *p < 0.05% versus control.

Chlorophyll fluorescence (Fig. 6I) and fresh biomass (Fig. 6II) were also analyzed. Analysis of the fluorescence of the dark-adapted sample versus maximum fluorescence (maximum PSII photosystem efficiency (Fv/Fm) made it possible to determine the relationship between the structure and function of the photosynthetic apparatus and to estimate the vitality of the plants. Shoot segments were adapted in the dark to ensure that all the reaction centers in photosystem II were opened (oxidized) and ready to receive electrons before the measurement. The highest chlorophyll fluorescence was characteristic of the series of plants grown on "Z" standard medium. The Fv/Fm level lower by 40% observed in the tap water variant indicates weaker efficiency of the PSII photosystem in the dark and a reduced demand for the macrophytes for products constituting the so-called assimilation strength under stress conditions, which was reflected in the growth of the studied plants. In the series with 1% leachate Fv/Fm was relatively high, (15% higher compared to the series grown on the tap water-based medium) which indicates high potential efficiency of photosystem II. Analysis of this physicochemical parameter indicates the possibility of using chlorophyll fluorescence as an indicator of plant damage caused by various environmental factors (especially salinity).

### Concentration of starch

**Figure 7:**
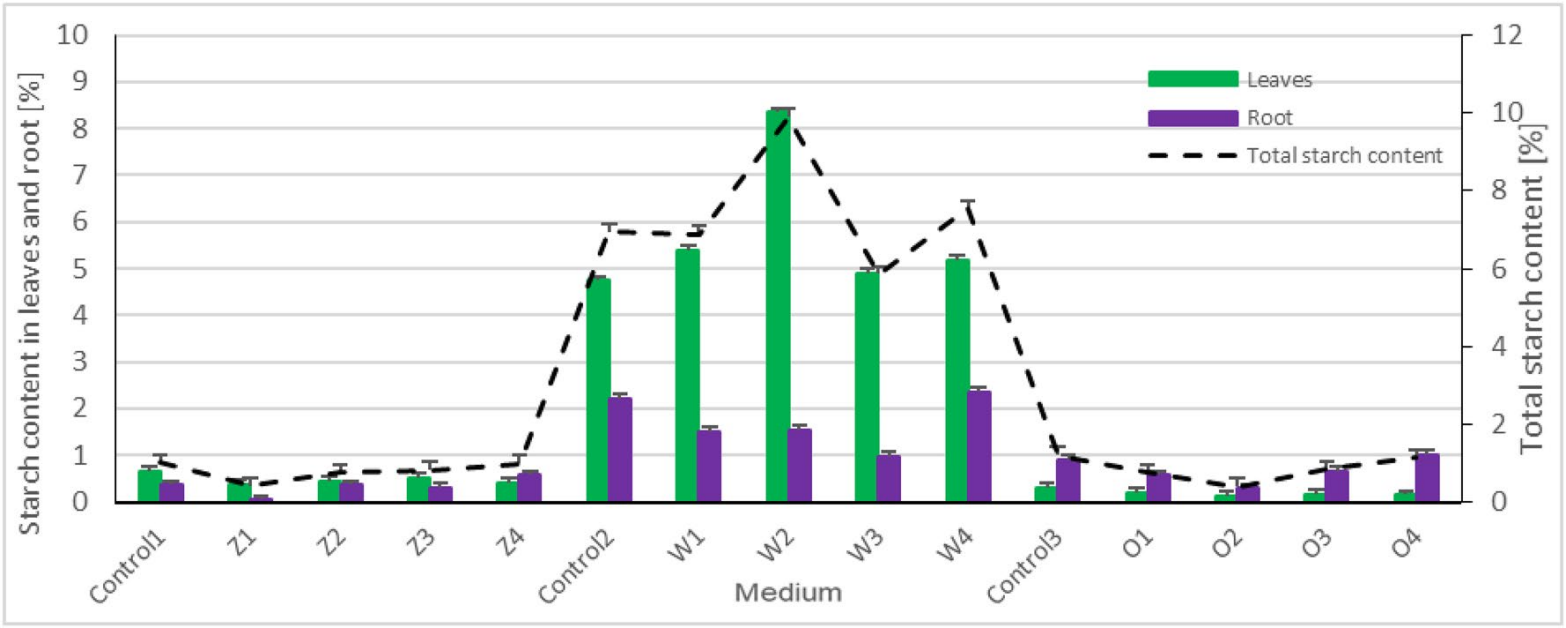
Total starch content in leaves and roots of *Spirodela polyrrhiza* plants grown on different variants of NaCl supplemented medium. Control series: Control 1–100 mL of "Z" medium; Control 2–100 mL of tap water; Control 3–99 mL tap water + 1 mL post-fermentation effluent; Experimental variants: "Z" medium supplemented with 25 mM NaCl (Z1), "Z" medium supplemented with 50 mM NaCl (Z2), "Z" medium supplemented with 75 mM NaCl (Z3), "Z" medium supplemented with 100 mM NaCl (Z4); tap water supplemented with 25 mM NaCl (W1), tap water supplemented with 50 mM NaCl (W2), tap water supplemented with 75 mM NaCl (W3), tap water supplemented with 100 mM NaCl (W4); 1% leachate supplemented with 25 mM NaCl (O1), water 1% leachate supplemented with 50 mM NaCl (O2), 1% leachate supplemented with 75 mM NaCl (O3), 1% leachate supplemented with 100 mM NaCl (O4). Data are expressed as mean ± SD of 10 experiments; Statistical analyses were performed using one-way parametric Anova test with the post-hoc Dunnett’s test. *p < 0.05% versus control.

The starch content (Fig. 7) in *Lemnaceae* plants was determined separately for shoots and root. Plants grown only on tap water and supplemented with NaCl (food stress–medium without nutrients and salt stress) were characterized by a high concentration of starch. In the W2 series (50 mM NaCl) compared to control (Control2) the starch level in the shoots was higher by 76% and in the roots lower by 30%. The starch contents in the other variants with tap water compared to the control (Control2) were as follows in the shoots: W1 (25 mM NaCl) higher by 14%; W3 (75 mM NaCl) higher by 3%; W4 (NaCl) higher by 10%; in the roots: W1 (25 mM NaCl) lower by 32%; W3 (75 mM NaCl) was lower by 56%; W4 (NaCl) higher by 7%.

The macrophytes grown on the medium with 1% post-fermentation effluent supplemented with NaCl compared to the control (Control3) were characterized by varying levels starch content in the shoots compared to the control for the O1 series (25 mM NaCl) was 34% lower; for O2 (50 mM NaCl) 61% lower; for O3 (75 mM NaCl) 42% lower; for O4 (100 mM NaCl) 51% lower. Starch content in the roots for the O1 series (25 mM NaCl) was 37% lower for O2 (50 mM NaCl) 69% lower; for O3 (75 mM NaCl) 27% lower; for O4 (100 mM NaCl) 10% higher.

*Spirodela* plants growing on "Z" standard medium with the addition of various concentrations of NaCl showed high sensitivity to salt stress, which was reflected in the level of starch production and storage in shoot segments. Compared to control (Control 1), the starch level in the shoots was lower and was as follows: for the Z1 series (25 mM NaCl) it was 42% lower; for Z2 (50 mM NaCl) it was 29% lower; for Z3 (75 mM NaCl) it was 21% lower; for Z4 (100 mM NaCl) it was 34% lower, in the roots: for Z1 series (25 mM NaCl) it was 89% lower for Z2 (50 mM NaCl) it was at the same level; for Z3 (75 mM NaCl) it was 11% lower, for Z4 (100 mM NaCl) it was 61% higher.

## Discussion

The presented results of our research relate to the result of the work of many authors, including Stelmach et al. (2021) and Szufa et al. (2019) who obtained interesting results in obtaining biomass from watercress. The authors of the above work obtained a biomass yield of 39.1–105.9 t ha^−1^ year^−1^ of water lash, and this was achieved using leachate as a nutrient source, and the physiological parameters determined in the plants were significantly higher than potential energy plants^42,50^. Like other authors, we also showed in our study that *S. polyrrhiza* has a high starch storage capacity under salt stress conditions^42,43^. Stelmach et al. (2021) and Wielgosinski et al. (2021) proved that adverse factors such as salt stress, sudden temperature drop, higher daily light intake (DLI) or nutrient deficiency, promoted starch production and accumulation. They proved that the starch content of watercress grown at 5 °C was 114% higher than that of watercress grown at 25 °C, and confirmed that photosynthetic efficiency depends on light availability (increase in DLI) and temperature. The results of their experiment indicated that starch production varied with temperature from 5 °C to 20 °C. The above authors, by changing the culture conditions from nutrient-poor medium to tap water, achieved an increase in starch content by 26.6%^51^. Similar results were obtained in our work using tap water as a medium supplemented with 50 mM NaCl. We obtained significantly higher starch compactness in shoot members compared to a series of plants grown on water alone. On the other hand, Toyama et al. (2018) studied four species of water cilia (*S. polyrrhiza, Lemna minor, Lemna gibba* and *Landoltia punctata*) and confirmed their ability to remove nitrogen from the environment^52^. They cultured these macrophytes for 4 days on municipal waste, animal husbandry waste and after anaerobic digestion. They proved that biomass production and nitrogen removal efficiency from all three types of liquid waste were most effective for *S. polyrrhiza*. Growth rates of all four species of water lash were higher on nitrogen-rich wastes, i.e. animal waste and anaerobic digestion wastes, than on municipal wastewater. It should also be noted that ethanol and methane production was higher from the biomass of *S. polyrrhiza* and *L. punctata* grown on each leachate compared to the other substrates^51,53^.

The ethanol production potential of some *Lemnaceae* species grown in ponds with leachate has been shown to be comparable to waste biomass (sugarcane), intercrops (alfalfa fiber)^47^, aquatic plants (water hyacinth and water lettuce)^54^ and *Lemna minor*^25,52^, *Wolffia globosa*^51,55^ or *Wolffia arrhiza*^56^, and microalgae such as *Chlamydomonas reinhardtii*^57^ and *Chlorella vulgaris*^28,57–59^. Our study confirmed that salt stress differentially affected the level of starch in the shoots and roots of *S. polyrrhiza* and was dependent on the concentration of NaCl.

In addition, macrophytes growing in polluted water bodies with high salinity levels can not only produce large amounts of biomass through starch storage, but can also reduce the level of salinity in the environment. Since *Lemnaceae* strains have been proven to have varying sensitivity to the amount of stored starch, strain optimization and ecotoxicological analyses of individual *Lemnaceae* strains are needed before they can be used on a large scale in the energy industry.

The results obtained in our study confirmed previous literature reports and indicated the relationship between starch accumulation in plants and the effect of salt stress on them. Similar findings were presented by Xu et al. (2011), who analyzed the effects of 10, 20 and 30 mM NaCl on plant growth, as well as on starch content, which was 13.4 and 18.7% higher than in the control sample, respectively^16^. On the other hand, Sree et al. (2015), in a study of four strains of *S. polyrrhiza*, showed a slight increase in the starch content of the cells, but in this experiment the level of NaCl used was much higher at 450 mM^18^.

Grajek et al. (2008) pointed out that access to low-cost starch and cellulosic feedstocks is crucial for bioethanol production. The development of innovative plant growth technologies for bioethanol production is a priority for the distilling and energy production industry^60^. Yin et al. (2015) analyzed biomass productivity in relation to bioethanol production and showed that it was not the quantity of biomass, but its quality, including plant starch content, light conditions and photoperiod, that was most important. It was shown that a 14-percent increase in grain yields due to fertilization with 120 kg Nxha^−1^ translated into increased ethanol production of 254.2 Liters per hectare^61^. *S. polyrrhiza*, on the other hand, could be a valuable feedstock for bioethanol production in the future due to its rapid biomass growth and high starch storage capacity^23,62^. However, the degree of starch production and accumulation depends on the species. In a study conducted by Ma et al. (2018) in 2017, different strains of *L. aequinoctialis* and *S. polyrrhiza* isolated from different latitudes were selected to determine their potential for bioethanol production^22^. The most favourable results were obtained with *L. aequinoctialis* 6000 plants, where biomass production was 15.38 ± 1.47 gm^−2^, starch content was 28.68 ± 1.10%, and starch production was 4.39 ± 0.25 gm^−2^. This strain had the highest starch production after 8 h of exposure, and tap water proved to be the most favorable substrate; interestingly, salt stress in the form of NaCl did not induce starch accumulation in plant cells.

Sree et al. (2016) using 16 varieties of macrophytes *Lemnaceae* indicated that salt stress had a positive effect reflected in increased starch accumulation in plants, however, some stressors (also NaCl) could inhibit the vegetative development of duckweed more effectively than a decrease in photosynthesis. Photosynthetic low carbohydrate production leads to low utilization by growth-related metabolism^17^. However, the varying range of plant responses to different concentrations of NaCl causing growth inhibition dependents on the species of plant and may indicate that macrophytes have alternative mechanisms for the use of excess energy.

Xu et al. (2011) used the two-step method to increase the starch content in *S. polyrrhiza* strain. First, the duckweed was grown under optimal conditions (nutrient-rich water) to produce a large amount of biomass, and then the plants were transferred to growth-limiting conditions (distilled water), resulting in the accumulation of starch. Sree et al. (2015) proposed an alternative one-step method of limiting the growth and stimulation of starch storage in water duckweed based on the use of salt stress in the range of 50 to 100 mM NaCl. Our results presented in this paper confirm these observations. In our experiment 50 mM NaCl most effectively stimulated starch accumulation in *S. polyrrhiza*.

In our laboratory experiments, we obtained 10% starch content (tap water plus a variant of 50 mM NaCl). We suggest that future experiments should take into account the different adaptive strategies of *Lemnaceae* to salt stress. The increased starch content observed in our experiment at lower NaCl levels, as in other studies, may be attributed to metabolic modification toward resistance or reduced toxicity of the stress factor. These processes may be related to the adaptive photosynthetic apparatus (mainly PSII), synthesis of various compounds, including glycerol, sugar, other osmoprotectants and specific proteins^14,52^. Increased NaCl in the medium also inhibits photosynthesis in *Wolffia arrhiza*, as evidenced by impaired electron transport and inactive photosystem II reaction centers^31^.

In our study, the decrease in the starch content in the roots was observed at the concentration of 100 mM NaCl. Salinity stress-induced inhibition of vegetative growth was reported for several duckweed species, including *S. polyrrhiza, L. minor and L. gibba*^17,18^. These observations may lead to the conclusion that the increase in salinity induces osmotic stress^26^ and activates the antioxidative plant defense system^63,64^. Similar effects were observed during starch analysis in plant roots^53^.

NaCl increased starch accumulation is mainly due to osmotic stress caused by primary stress. Plant responses to osmotic stress are regulated by abscisic acid (ABA). Liu et al. (2018) proved that high biomass growth and starch accumulation in *L. punctata* plants were caused by abscisic acid^53^. ABA supplementation increased the percentage of starch from 2.29% to 46.18% after 14 days, with a total starch level 2.6-fold higher compared to the control group. They showed that endogenous ABA increased biomass efficiency production and promoted the accumulation of starch by duckweed, but also affected the level of other endogenous hormones, it increased zeatin riboside and indole-3-acetic acid, and decreased gibberellin. In addition, abscisic acid regulated the activity of enzymes involved in starch biosynthesis and duckweed catabolism. The activity of enzymes involved in starch biosynthesis increased, while the activity of enzymes catalyzing starch degradation decreased after using ABA. Liu et al. (2018) concluded that ABA might promote biomass and starch accumulation regulating endogenous hormone levels and the activity of key enzymes associated with starch metabolism^53^.

Rapid growth of high-quality duckweed biomass is considered a key step to its use for the production of biofuels. Under ideal conditions, water duckweed could double its biomass in 16–48 h. The rate of growth of water duckweed can reach 12.4 g/m^2^/day of dry matter, and its efficiency was documented at 55 tons/ha/dry weight^26^, so much higher compared to the energy plants used so far.

**Figure 8:**
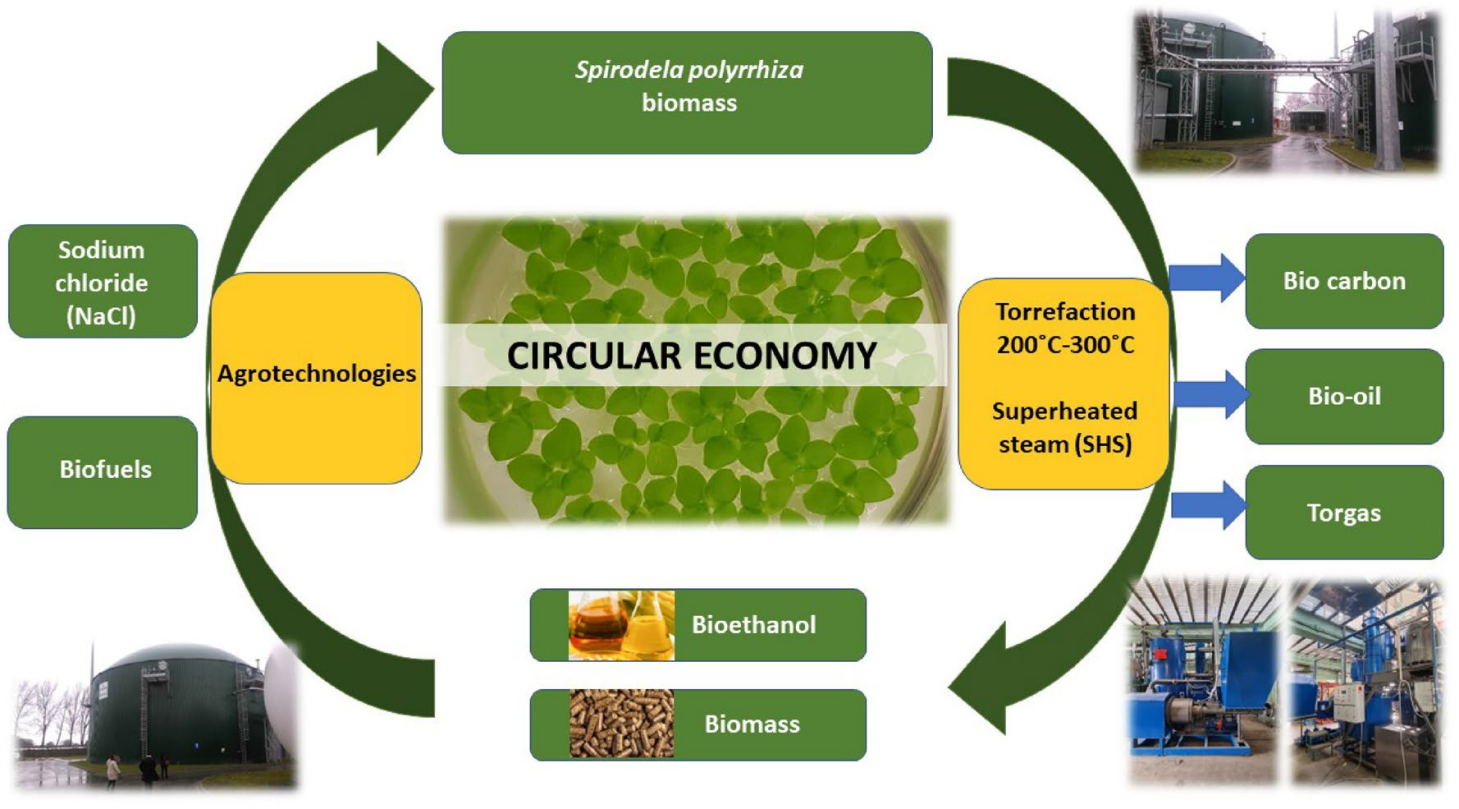
Circular economy concept for valorization of *Spirodela polyrrhiza* biomass to produce different products: solid and liquid biofuels.

The experiments showed multidirectional productivity of aquatic plants *Lemnaceae* Fig. 8. High biomass content in the plants cultivated on the medium supplemented with postfermentation leachate indicated the possibility of using effluent from biogas plant. The use of leachate in the designed cost-efficient culture medium increases profitability and competitiveness of a biorefinery with regard to ecologic and environmental issues. Management of biogas plants wastes will allow to diminish ecological footprint of this type of installations and to significantly decrease bioethanol production costs.

All-year vegetation period, quick and high biomass yield, cost-efficient production and high starch content make water plant *Lemnaceae* an alternative to the plants now used for bioethanol production (corn, cereals, sugar beet). Production of energy plants poses many problems including limitation of the area for their growth, harmful effect of adverse weather conditions, high water consumption. The production of the water plants *Lemnaceae* with the use of biogas plant waste meets the requirements of sustainable technology of renewable energy resources and sustainable recycling economy^4,58,65^.

The high (up to 70%) starch content and low content of lignin and hemicellulose in *Lemnaceae* as compared to the other energy plants, from the technological point of view is much more effectively used for bioethanol production. They do not require preliminary heat treatment, which in standard procedures is time consuming and costly. *Lemnaceae* biomass is produced in bioreactors or in open water bodies. It can be used as a multi-protein supplement of animal feed, as a bioindicator to assess environmental pollution and in phytoremediation during processing of municipal and agricultural waste.

The obtained results indicated that *Spirodela sp.* in response to abiotic stress such as high salinity of the environment was able to increase starch production in comparison with other popular energy plants. The use of duckweed as a source of starch for the production of bioethanol allows to create a wide spectrum of new possibilities for this group of plants. High accumulation of starch with low lignin content increased ability to absorb nutrients such as nitrogen and phosphorus from leachate, increased absorption of CO_2_ due to intensified photosynthesis make *S. polyrrhiza*, similarly as other water duckweeds, a promising raw material for the production of biofuels^66^. Our experiment confirms the effective use of aquatic plants in the production of biofuels, which creates opportunities for the development of innovative, alternative and cost-effective energy sources, and is confirmed by the 2023 review publication.

## Conclusion

In recent years, scientific advances in the energy sector have fundamentally contributed to the development of several environmentally friendly technologies and processes, particularly in the production of biofuels including bioethanol. These solutions allow the production of biofuels from plant biomass and the development of clean green technologies. In recent years, there has been a growing interest in the production of bioethanol as a liquid fuel for powering motor vehicles. Ethanol can be produced from products containing monosaccharides as well as polysaccharides including starch and represent promising feedstocks for larger-scale bioethanol production. Technological advances are enabling improved production techniques and cost reductions that will favor the production of bioethanol from *S. polyrrhiza* plants as a fuel, especially in view of the benefits that its potential production entails in terms of less environmental contamination, agricultural activation and reduced oil import costs.

## Data Availability

The manuscript provides averages from many experiments, which accurately demonstrate the effect of the NaCl applied on the development and physiological activity of *Spirodela polyrrhiza*. The presented results give grounds for their use in practice in the torification process. Data show the important information for science in the field the potential to increase the starch content and energy performance of *Spirodela* response to the applied treatments. However, the detailed results will be used by the authors for further studies, which will constitute the content of the patent. For commercial reasons, contractors do not agree to share detailed results that would enable their direct use in similar conditions. Zdzislawa Romanowska-Duda (zdzislawa.romanowska@biol.uni.lodz.pl) can be contacted.
